# Epidemiological analysis of congenital syphilis in the State of Paraná, Brazil

**DOI:** 10.1590/1806-9282.20231170

**Published:** 2024-05-03

**Authors:** Fabio Vinicius Barth, João Pedro Gambetta Polay, Camila Ost

**Affiliations:** 1 Universidade Estadual de Ponta Grossa, Department of Medicine – Ponta Grossa (PR), Brazil.; 2 Universidade Estadual de Ponta Grossa, Maternal and Infant University Hospital – Ponta Grossa (PR), Brazil.

**Keywords:** Epidemiology, Syphilis, Congenital, Maternal-child health services

## Abstract

**INTRODUCTION:**

Congenital syphilis is a complex public health issue caused by the transmission of *Treponema pallidum*. Brazil has high incidence rates, with a distinct transmission pattern surpassing other notifiable diseases.

**OBJECTIVE:**

The objective of this study was to examine epidemiological trends, incidence rate, mortality, geographical distribution, prenatal care, and diagnostic determination timing of congenital syphilis in Paraná State.

**METHODS:**

Data from Department of Informatics of the Single Health System were used to analyze the period from 2015 to 2021 in Paraná. Linear regression and t-tests were employed to assess significance. Statistical significance was determined by p<0.05.

**RESULTS:**

A total of 5,096 notifications of congenital syphilis were recorded in Paraná over the examined period. The metropolitan region is a notable clustering of cases, following Londrina, Maringá, and Foz do Iguaçu. The age group with the highest cases is found between 20 and 24 years (34.93%). Regarding maternal education, a higher occurrence was noticed in incomplete lower secondary education mothers (22.12%). Regarding ethnic background, 3,792 women were identified as white, which was the majority of this analysis (74.41%). Diagnosed maternal syphilis throughout the prenatal phase during 2015–2018 exhibited a noteworthy increase (p<0.05). Most women received prenatal care (p<0.05), even though a significant number received the diagnosis at the delivery or after it. The average infant mortality rate associated with congenital syphilis in Paraná was 0.03.

**CONCLUSION:**

Paraná State serves as a representative sample of this epidemiological situation, providing significant insights into the intricacies of congenital syphilis incidence. Further comparative investigations including diverse regions within Brazil are necessary.

## INTRODUCTION

Congenital syphilis remains a difficult and challenging issue in the field of global public health, as it is caused by the vertical transmission of the etiological agent, *Treponema pallidum*. The illness in question is characterized by a complex interplay of biological, social, and health-related elements, resulting in notable resilience despite advancements in medical and technology interventions^1,2^.

In the specific context of Brazil, this disorder assumes a very concerning magnitude, considering the documented high incidence. Congenital syphilis exhibits a distinctive pattern of transmission, characterized by infection rates that frequently surpass those of other illnesses that are subject to mandatory reporting. The reports have been conducted by thoroughly examining and analyzing data by the Department of Informatics of the Single Health System (DATASUS), which has been prepared by the Notification Accidents Information System (SINAN). The aforementioned departments facilitate research efforts aimed at delineating the epidemiological panorama of congenital syphilis, hence enabling the detection of patterns and the development of focused treatments^2^.

At the national level, a more comprehensive understanding of the intricacies of congenital syphilis emerges when we examine the broader context. The intricate nature of disease transmission is inherently intertwined with systemic factors and the vulnerability of healthcare systems. The dearth of high-quality prenatal care and inadequate allocation of resources for the advancement of sexual and reproductive health education are critical determinants^3^.

The issue of congenital syphilis extends beyond the borders of Brazil and has global implications, irrespective of a country's level of development. The persistence of this illness in both developed and developing nations underscores the existing disparities in health systems and the obstacles that hinder access to reproductive health treatments. Insufficient access to education and less knowledge on the prevention of vertical transmission exacerbate the current circumstances^4,5^. According to the World Health Organization, the establishment of diagnostic, therapeutic, and preventive guidelines for congenital syphilis aims to foster a worldwide alliance in combating this ailment^6^.

The thorough analysis of the incidence of congenital syphilis in the state of Paraná yields a significant and insightful viewpoint. The comprehensive examination of the data accessible in SINAN and Information System in Live Births (SINASC), facilitated by DATASUS, has the potential to unveil distinct geographical, demographic, and temporal trends pertaining to this location. This information might provide valuable insights for the development of preventative and control methods tailored to the specific local context. By comprehending congenital syphilis in this framework, it becomes feasible to tackle not only the clinical facets but also the social and behavioral factors that impact the transmission of the ailment^3^.

The main aim of this study was to conduct a comprehensive and evidence-based examination of the prevalence of congenital syphilis in the state of Paraná, focusing on the period from 2015 to 2021. The study was grounded in the recognition that Paraná serves as a representative sample of this epidemiological situation, providing significant insights into the intricacies of congenital syphilis incidence and the potential consequences of its determinants in specific places.

## METHODS

This study was conducted as a transversal quantitative research. The collection of data on recorded cases of congenital syphilis was conducted by leveraging the information maintained within SINAN and SINASC, which covers Paraná's geographical region. The dataset covers the period from January 2015 to December 2021. The factors that were examined in this study include the rate of incidence, infant age at the time of diagnosis, the technique utilized for a definite diagnosis, death instances, provision of prenatal care, and the diagnostic determination timing.

The data were systematically arranged in tabular format, by using the Microsoft Excel software, encompassing both absolute numerical values and corresponding relative percentages. The analysis involved an investigation of the variables and their distribution throughout the years under consideration. There was no need to provide the assessment report to the Research Ethics Committee due to the utilization of publicly available data.

The statistical analysis was conducted using the GraphPad Prism 10 software (GraphPad Software Inc., San Diego, CA, USA). The incidence data were analyzed using simple linear regression to determine the slopes, standard error, R-squared, and p-value. The t-test was employed to analyze variance. The 95% confidence interval was computed for each trend and determined to have a significant level (p-value) of 0.05. A p<0.05 was the threshold for statistical significance. The results obtained were subsequently presented.

## RESULTS

A total of 5,096 notifications of congenital syphilis were recorded in the state of Paraná over the examined period, utilizing the secondary data provided by the DATASUS platform. In terms of the yearly distribution, there was a discernible decline in the incidence of the disease, as evidenced by 867 diagnoses in 2019 and 351 cases in 2021, which, respectively, marked the greatest and lowest recorded values over the past decade.

When examining the partitioning of the Paraná State into Health Regions, it becomes apparent that there is a notable clustering of cases in Curitiba and the surrounding metropolitan region, which constitutes the second health region. These areas collectively accounted for a total of 2,282 cases over the specified time of inquiry. The Health areas of Londrina, Maringá, and Ponta Grossa, which have dense populations, have elevated notification rates, with corresponding case counts of 509, 445, and 214. It is noteworthy to mention the significant occurrence of cases in the Paranaguá region (239 cases) and the Foz do Iguaçu region (341 cases), with the former being a port area and the latter serving as a border zone between Argentina and Paraguay. The high prevalence of syphilis in some regions can be attributed to the substantial movement of individuals in these locations, as syphilis is mostly spread through sexual contact^5,7^.

**Figure 1:**
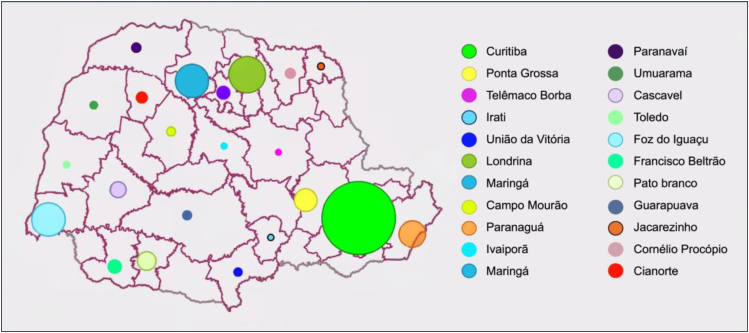
Map displaying circles distributed proportionally to the notifications of congenital syphilis cases in each health region in the state of Paraná. Source: the authors.

Furthermore, it has been observed that health areas located at a considerable distance from major metropolitan centers demonstrate the lowest rates of congenital syphilis. Notable examples include the areas of Irati (with 12 reported cases), Jacarezinho (with 22 reported cases), and Ivaiporã (with 23 reported cases). The observed gap may be ascribed to a diminished population contingent in these places or the potential underreporting of the analyzed cases^7,8^. Figure 1 graphically depicts the spatial distribution of cases within the state of Paraná through the utilization of a map of circles.

The analysis regarding the maternal age group has demonstrated its substantial influence on the incidence of congenital syphilis, as shown in Table 1. The age group between 20 and 24 years has the highest concentration of people (34.93%) followed by the age group between 25 and 29 years (22.12%). On the contrary, the obtained data showed that the maternal age groups at the extremes had the lowest proportions of confirmed cases in neonates. The degree of maternal education is an additional factor that contributes to the risk of *T. pallidum* bacterial transmission^7^.

**Table 1:** Analysis of the maternal epidemiological profile of reported cases of congenital syphilis.

Variants		n	%	95%CI	p-value
Mother's level of education					
	Ignored/null	1,058	20.76	76.00–215.00	<0.0001
	No schooling	45	0.88	2.00–17.00	<0.0001
	Incomplete primary	207	4.06	16.00–36.00	<0.0001
	Complete primary	151	2.96	8.00–30.00	<0.0001
	Incomplete lower secondary	1,084	21.27	69.00–214.00	<0.0001
	Complete lower secondary	655	12.85	41.00–135.00	<0.0001
	Incomplete higher secondary	739	14.50	54.00–156.00	<0.0001
	Complete higher secondary	959	18.82	74.00–189.00	<0.0001
	Incomplete university	91	1.79	7.00–23.00	<0.0001
	Complete university	77	1.51	2.00–17.00	<0.0001
	Not applied	30	0.59	2.00–7.00	<0.0001
	Total	5,096			
Mother's age group (years)					
	Ignored/null	51	1.00	1.00–17.00	<0.0001
	10–14	33	0.65	1.00–9.000	<0.0001
	15–19	1,033	20.27	57.00–196.00	<0.0001
	20–24	1,780	34.93	129.00–316.00	<0.0001
	25–29	1,127	22.12	87.00–204.00	<0.0001
	30–34	588	11.54	37.00–105.00	<0.0001
	35–39	366	7.18	24.00–65.00	<0.0001
	40–44	109	2.14	10.00–21.00	<0.0001
	45–49	9	0.18	0.00–3.00	<0.0001
	Total	5,096			
Mother's ethnicity					
	White	3,792	74.41	408.70–674.80	<0.0001
	Black	104	2.04	11.30–18.42	<0.0001
	Yellow	16	0.31	1.41–3.16	<0.0001
	Mixed	743	14.58	71.41–140.90	<0.0001
	Indigenous	12	0.24	3.09	<0.0001
	Ignored	429	8.42	42.81–74.33	<0.0001
	Total	5,096			

Source: the authors.

The findings of the analysis suggest that there was a higher occurrence of the mentioned incidence in infants born from mothers with incomplete lower secondary education (21.27%). This was followed by mothers who completed higher secondary education (18.82%), those who did not finish higher secondary education (14.50%), and those who completed lower secondary education (12.85%). On the contrary, it is noteworthy that the proportion of women who have attained a higher level of education, such as university level, for example, either completed or partial, is just 3.30% of the total instances, as depicted in Table 1. The correlation between younger age groups and lower educational level is associated with risk behavior, hence facilitating the transmission of the disease^7^.

The ethnic background of the mother might offer significant insights into identifying the factors that contribute to the risk linked with the condition. This investigation identified 3,792 women as belonging to the white ethnic group. Additionally, the analysis identified 743 women as mixed, 104 as black, 16 as yellow, and 12 as indigenous. However, information for 429 cases was unavailable.

Regarding the monitoring of pregnant individuals and the timing of congenital syphilis notification, Table 2 provides a comprehensive overview of the trends observed between 2015 and 2021. This table illustrates the various stages of diagnosis, such as prenatal care, delivery/curettage, and the postpartum periods, shedding light on the intricacies associated with these instances. It was comprehended by a thorough individual analysis of each instance that the occurrence of maternal syphilis throughout the prenatal phase exhibited a noteworthy increase (p<0.05) of occurrences from 2015 to 2018. Upon analyzing the occurrence of maternal syphilis after labor or curettage, no statistically significant increases or decreases in cases were observed (p>0.05). With respect to the occurrence of maternal syphilis following delivery, the data presented in Table 2 do not indicate any statistically significant changes in the number of cases, as demonstrated by a p>0.05.

**Table 2:** Epidemiological linear regression analysis of congenital syphilis cases in Paraná from 2015 to 2021, including maternal notification, incidence during prenatal care, at delivery, after delivery, and infant's mortality incidence.

Variants					
Incidence of diagnosed women who underwent prenatal care during 2015–2021					
Realized prenatal diagnosis	Confirmed cases (n)	%	p-value		
No	54	1.06	0.003216		
Yes	4,560	89.48			
Ignored	482	9.46			
Total	5,096				
Diagnosis during prenatal care					
Period	Y-intercept	X-intercept	95%CI	p-value	R^2^
2015–2021	50,198	2,040	–95.54 to 46.33	0.4134	0.284
2015–2018	-117787	2,007	31.21–86.19	0.0116	0.9768
2018–2021	271,345	2,023	-340.60 to 72.38	0.1078	0.7961
At the time of delivery/curettage					
2015–2021	7,775	2,054	-22.36 to 14.78	0.6226	0.05206
2015–2018	-37565	2,009	-16.10 to 53.50	0.147	0.7277
2018–2021	57,690	2,024	-81.96 to 24.96	0.1488	0.7246
After delivery					
2015–2021	7,243	2,028	-7.97 to 0.8292	0.0913	0.4654
2015–2017	-12051	2,009	-1.33 to 13.34	0.0611	0.9908
2017–2021	12,551	2,024	-15.87 to 3.467	0.1339	0.5814

Source: the authors.

Moreover, when considering the frequency of diagnosed women who received prenatal care, as depicted in Table 2, it becomes apparent that a significant majority of diagnoses in pregnant women occurred during the prenatal period (4,560 cases). This finding demonstrates a statistically significant difference (p<0.05) between the group of women who underwent prenatal treatment and those who did not. Therefore, the results show that a lot of the pregnant women who were diagnosed with congenital syphilis had received prenatal care. This is different from cases that were found during childbirth or curettage procedures, as well as in the time after giving birth. Consequently, additional investigations are warranted to elucidate the factors contributing to the delayed diagnosis of these patients during the prenatal stage. Moreover, there is a required critique regarding the significance of systematic monitoring of pregnant women and the administration of syphilis detection tests throughout the gestational period and during delivery or curettage procedures in cases of abortions^9^.

According to the guidelines established by the Ministry of Health of Brazil, it is suggested to perform serological testing for syphilis during prenatal visits, irrespective of the maternal illness history^9-11^. In Paraná, the data reveal that prenatal care was not received by nearly 10% of pregnant mothers (482 out of 4,543) whose infants were diagnosed with congenital syphilis, excluding instances with missing information. The assessment of the fetus before birth is of utmost importance in facilitating appropriate fetal growth, identifying disorders in the gestational period, and implementing timely interventions to impede the advancement of these ailments^3,10,11^.

The assessment of disease incidence can be informed by analyzing death rates associated with congenital syphilis. During the investigation period in Paraná, a total of 37 deaths officially related to this particular illness were registered. The distribution of fatalities with respect to age groups, as shown in Table 2, is as follows: there were 25 fatalities recorded within the initial 6 days of life, followed by four deaths between 7 and 27th days, with an additional eight deaths occurring from 28 to 364th days of life. Moreover, based on the information that was available, it was feasible to compute the yearly mortality rate utilizing the SINASC data of the state of Paraná, encompassing the period from 2015 to 2021. The findings of the study indicate that the average infant mortality rate associated with congenital syphilis in the state of Paraná for the period 2015–2021 was 0.03 deaths per 1,000 live births. With regard to the yearly rate, it is worth noting that in the year 2015, there was a rate of 0.05; however, in the year 2021, the rate was recorded at 0.01.

The incidence of congenital syphilis cases in Paraná, totaling 5,096 records, appears to be significantly high when contrasted with epidemiological data from the surrounding states in the southern region of Brazil. In comparison, Santa Catarina had a lower number of cases, totaling 3,866, while Rio Grande do Sul recorded the highest incidence with 11,953 cases^2^. Such disparities indicate epidemiological variations within the region, suggesting the need for more in-depth analyses to understand the factors determining these discrepancies and implement effective strategies for preventing and controlling congenital syphilis^12,13^.

## CONCLUSION

The results pertaining to the epidemiological characteristics of congenital syphilis in the state of Paraná align with the guidelines established by the Ministry of Health for the prevention, diagnosis, and treatment of this ailment. The analysis of the data indicates that among the main factors contributing to the progression of more severe manifestations of the disease and mortality are delayed diagnosis and insufficient treatment. This underscores the critical need for prenatal monitoring and administering non-treponemic testing to pregnant mothers and babies as a preventative intervention. The imperative of promoting sexual education arises as crucial in the effort to mitigate the incidence of sexually transmitted illnesses.

In the context of a prospective study on congenital syphilis, it is recommended to do further comparative investigations including diverse regions within Brazil. These studies would offer a complete perspective on the condition at a national scale, taking into account the public health network's ability to provide care to the susceptible population affected by this disease.
